# MAT2A facilitates PDCD6 methylation and promotes cell growth under glucose deprivation in cervical cancer

**DOI:** 10.1038/s41420-022-00987-6

**Published:** 2022-04-08

**Authors:** Hui Luo, Yizuo Song, Jian-an Zhang, Yi Liu, Fengyun Chen, Zhiwei Wang, Xueqiong Zhu

**Affiliations:** Center for Uterine Cancer Diagnosis & Therapy Research of Zhejiang Province. Department of Obstetrics and Gynecology, the Second Affiliated Hospital of Wenzhou Medical University, Wenzhou, 325027 Zhejiang China

**Keywords:** Cervical cancer, Cervical cancer

## Abstract

The underlying mechanisms of methionine adenosyltransferase 2 A (MAT2A)-mediated cervical cancer progression under nutrient stress are largely elusive. Therefore, our study aims to investigate molecular mechanism by which MAT2A-indcued cervical oncogenesis. The interaction between MAT2A and programmed cell death protein 6 (PDCD6) in cervical cancer cell lines was detected by immunoprecipitation, immunoblotting and mass spectrometric analysis. A panel of inhibitors that are linked to stress responsive kinases were utilized to detect related pathways by immunoblotting. Cell proliferation and apoptosis were investigated by CCK-8 and flow cytometry. Apoptosis related protein level of Bcl-2, Bax and Caspase-3 was also analyzed in cells with PDCD6 K90 methylation mutation. The association between MAT2A and PDCD6 was detected by immunohistochemistry and clinicopathological characteristics were further analyzed. We found that the interaction between MAT2A and PDCD6 is mediated by AMPK activation and facilitates PDCD6 K90 methylation and further promotes protein stability of PDCD6. Physiologically, expression of PDCD6 K90R leads to increased apoptosis and thus suppresses growth of cervical cancer cells under glucose deprivation. Furthermore, the clinical analysis indicates that the MAT2A protein level is positively associated with the PDCD6 level, and the high level of PDCD6 significantly correlates with poor prognosis and advanced stages of cervical cancer patients. We conclude that MAT2A facilitates PDCD6 methylation to promote cervical cancer growth under glucose deprivation, suggesting the regulatory role of MAT2A in cellular response to nutrient stress and cervical cancer progression.

## Introduction

Cervical cancer ranked 4^th^ cause of cancer-related death among women in the world, with the reported new cases and deaths reaching 604,127 and 341,831 in 2021, respectively [1]. The main cause of cervical cancer development is the human papillomavirus (HPV) infection [2]. The metabolic reprogramming is also critically involved in tumorigenesis of human cancers. Specially, activation of metabolic reprogramming in cancer often leads to enhanced nutrient uptake to supply energetic pathways. However, nutrient limitations within solid tumors may be required because cancer cells display metabolic flexibility to sustain growth [3, 4]. Hence, a better understanding of its dynamics may be essential for developing new therapeutic targets and treatment strategies for cervical cancer.

Growing studies have revealed that metabolic enzymes closely regulate protein post-translational modification (PTM) through the catalytic activity for metabolite production [5]. Methionine adenosyltransferase 2 A (MAT2A) is an essential regulator in cellular metabolism and catalyzes the reaction of L-methionine and adenosine triphosphate (ATP) to S-adenosylmethionine (SAM) [6]. SAM acts as the major methyl donor required for methyltransferases-mediated methylation of DNA, RNA and proteins to fundamentally regulate cellular activities [7], which is also involved in other metabolic pathways. To date, accumulated evidence has identified the tumorigenic role of MAT2A in several human cancers, including colon cancer [8], human hepatocellular carcinoma [9], gastric cancer [10] and breast cancer [11].

Programmed cell death protein 6 (PDCD6) is a 22 kDa calcium-binding protein comprising five serially repetitive EF-hand structures. PDCD6 participates in T-cell receptor-, Fas-, and glucocorticoid- induced programmed cell death [12, 13]. Recent studies reveal a dual expression pattern of PDCD6 in human cancers in a tissue-specific manner. For instance, PDCD6 was upregulated in lung cancer [14], hepatomas [14] and metastatic ovarian cancer [15], while PDCD6 was decreased in non-small cell lung cancer [16] and gastric cancer [17]. However, studies focusing on the role of PDCD6 in human cervical cancer are still rare.

The capacity to sense and respond to nutrient availability is essential for cells to modulate normal cellular functions [18]. Notably, cancer cells often face with numerous metabolic conditions, such as nutrient shortage including glucose and oxygen [19]. Remodeling in metabolic network and nutrient utilization enables cancer cells to adapt and survive under the nutrient scarcity [18]. Particularly, glucose starvation is one of the major forms of metabolic stress in cancer cells, which favors anaerobic glycolysis (also known as Warburg effect) to generate over 50% of cellular ATP [20]. During this process, the glucose level in the tumor microenvironment is further reduced due to increased glucose consumption and limited supplies. in [21]. However, the role of glucose deprivation-mediated protein methylation in cervical cancer remains undefined.

In the present study, we investigated the interaction between MAT2A and PDCD6 under glucose deprivation and the underlying signaling pathway. We also explored the effects of MAT2A and PDCD6 on cell viability and apoptosis in cervical cancer cells. PDCD6 methylation mutation cells were also structured to detect the most important methylation cite, which promotes PDCD6 methylation and protein stability. Moreover, the protein level of MAT2A and PDCD6 in cervical cancer patients was validated using IHC analyses, and the clinicopathological characteristics were also analyzed. Our study will provide a novel mechanism by which MAT2A facilitates methylation of PDCD6 to promote cervical cancer growth, suggesting that MAT2A and PDCD6 might be the targets for cervical cancer therapy.

## Results

### MAT2A interacts with PDCD6 under glucose deficiency

**Fig. 1:**
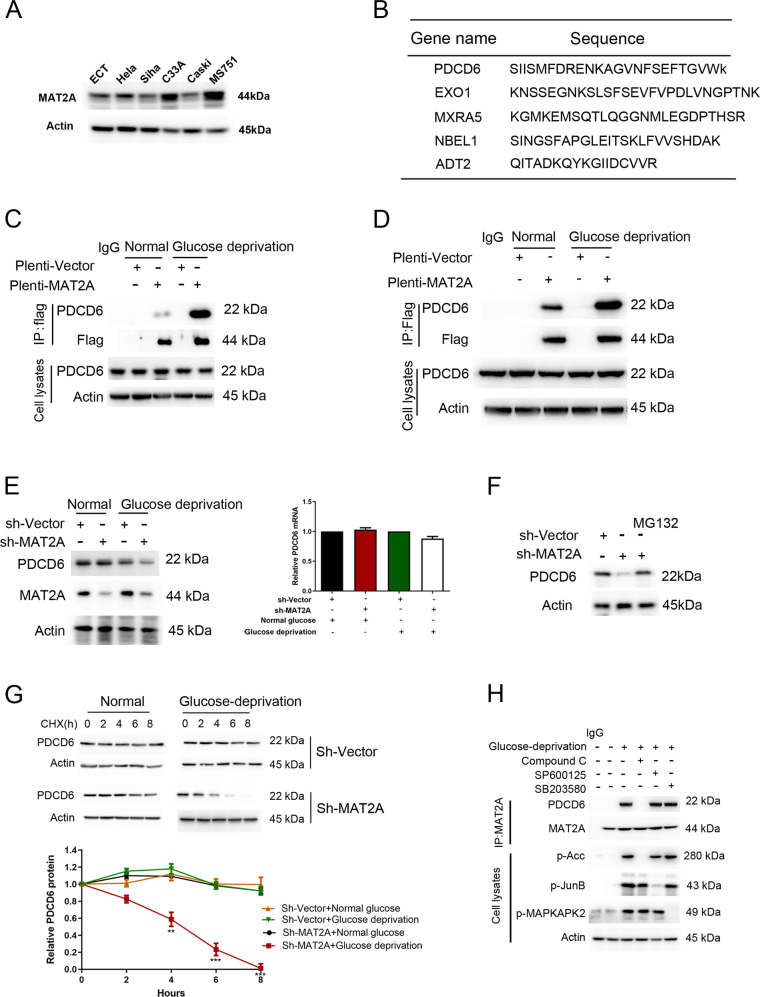
Glucose deficiency induces MAT2A-PDCD6 interaction. **A** Protein levels of MAT2A were detected by immunoblotting in normal cervical epithelial cells and cervical cancer cell lines. **B** The mass spectrometry analysis was performed in Flag tagged-MAT2A cell. MAT2A-associated proteins were listed. **C** MS751 cells transfected with or without 3×flag-MAT2A were cultured for 12 h under normal or glucose deprivation. Cellular extracts were subjected to immunoprecipitation with an anti-PDCD6 antibody. **D** C33A cells transfected with or without 3×flag-MAT2A were cultured for 12 h under normal or glucose deprivation. Cellular extracts were subjected to immunoprecipitation with an anti-PDCD6 antibody. **E** MS751 cells transfected with or without MAT2A shRNA were cultured for 12 h under normal or glucose deprivation. Western blotting analysis was used to determine MAT2A protein and PDCD6 protein levels (Left panel). Relative mRNA expression of PDCD6 was analyzed using RT-PCR (Right panel). **F** MS751 cells transfected with or without MAT2A shRNA were cultured for 12 h. The MS751 cells with MAT2A shRNA were treated with 10 μM MG132 for 6 h. Cell lysates were analyzed by Western blotting. **G** MS751 cells with stable expressing wild-type MAT2A or sh-MAT2A were cultured in glucose deprivation condition for 12 h, and then CHX (10 μg/ml) treatment was applied for different time courses (0, 2, 4, 6, 8 h) before harvest. The below panel showcases relative protein amounts of different groups. Error bar mean ± s.d. of triplicate experiments. ^***^P < 0.001, ^**^P < 0.01, ^*^P < 0.05. **H** MS751 cells with expressing Flag-MAT2A were pretreated with Compound C (10 μM), SP600125 (20 μM) and SB203580 (10 μM) for 1 h before being cultured with glucose deprivation for 12 h. Cellular extracts were subjected to immunoprecipitation with an anti-MAT2A antibody. Cell lysates were directly subjected to Western blotting.

To determine the potential functional effect of MAT2A on cervical cancer cells, MAT2A protein expression was first examined across a panel of human cervical cell lines. A higher level of MAT2A protein was detected in MS751 and C33A cells compared with that in normal cervical epithelial cells (ECT1) and other cervical cancer cell lines (Fig. 1A). Hence, MS751 and C33A cell lines were selected for further experiments. Subsequently, Flag tagged-MAT2A was stably expressed in MS751 cells, and the mass spectrometry analysis was performed after the immunoprecipitation using the anti-Flag antibody to identify MAT2A-associated proteins. According to the enrichment, we selected five genes that were significantly enriched interactors of MAT2A (Fig. 1B). The interaction between MAT2A and PDCD6 was verified by co-immunoprecipitation analysis (Fig. 1C). More importantly, glucose deprivation treatment largely enhanced MAT2A-PDCD6 complex formation both in MS751 (Fig. 1C) and C33A cells (Fig. 1D), suggesting that MAT2A-PDCD6 interaction was responsive to nutrient stress.

### AMPK promotes MAT2A-PDCD6 interaction

To investigate whether MAT2A affects PDCD6 protein status, MAT2A was depleted in MS751 cells by a validated shRNA. Specifically, MAT2A depletion resulted in a decreased PDCD6 protein expression (Fig. 1E, left panel) but not PDCD6 mRNA (Fig. 1E, right panel) level under glucose deprivation. This change was reversed by the protease inhibitor MG132 treatment (Fig. 1F), suggesting that MAT2A was required for PDCD6 protein stability maintenance. Consistent with this notion, the half-life of PDCD6 was dramatically reduced by MAT2A depletion under glucose deprivation instead of normal condition (Fig. 1G). For exploring the underlying mechanism that regulated the MAT2A-PDCD6 interaction, several stress responsive kinases inhibitors were used. It was found that MAT2A-PDCD6 complex formation under glucose deprivation was abrogated by treatment of the AMPK inhibitor compound C but not the JNK inhibitor SP600125 or the p38 inhibitor SB203580 in MS751 cells (Fig. 1H). These data indicated that AMPK activity was required for MAT2A binding to PDCD6, and maintained the stability of PDCD6 protein under glucose-deficient condition.

### PDCD6 counteracts apoptosis of cervical cancer cells under glucose deprivation

**Fig. 2:**
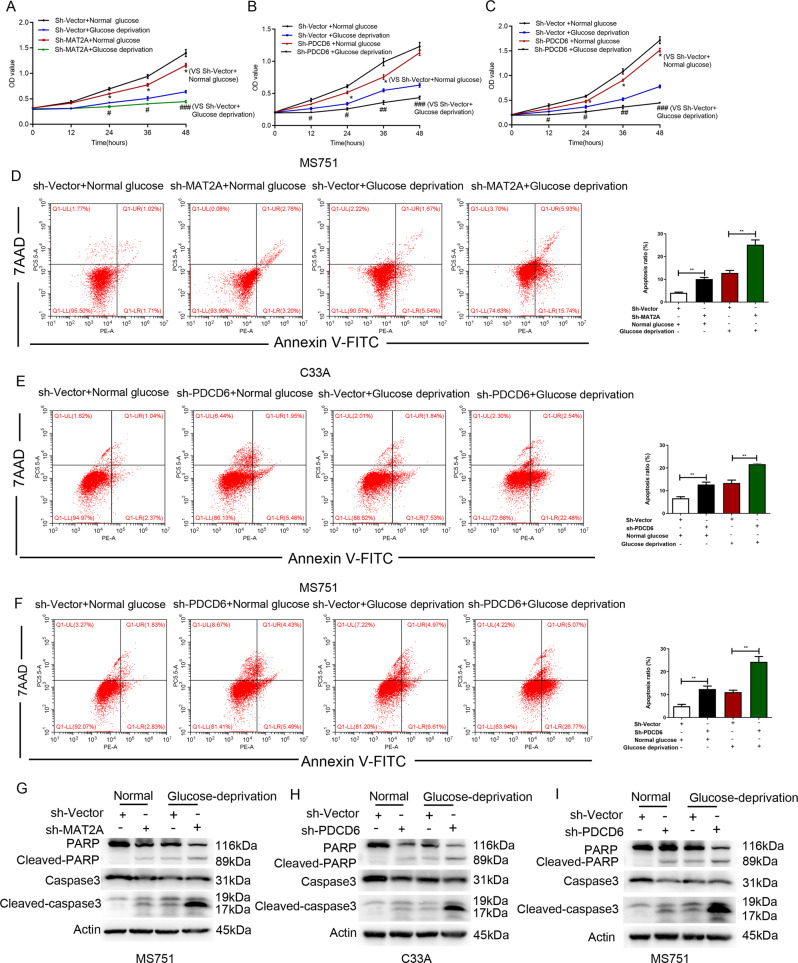
PDCD6 promotes proliferation and inhibits apoptosis of MS751 cells. **A** The proliferation of MS751 cells with or without MAT2A depletion was examined by CCK8 assay under normal or glucose deprivation. Error bar mean ± s.d. ^*^P < 0.05. ^###^*P* < 0.001, ^#^*P* < 0.05. **B** The proliferation of C33A cells with or without PDCD6 depletion was examined by CCK8 assay under normal or glucose deprivation. Error bar mean ± s.d. ^*^P < 0.05. ^###^*P* < 0.001, ^##^*P* < 0.01, ^#^*P* < 0.05. **C** The proliferation of MS751 cells with or without PDCD6 depletion was examined by CCK8 assay under normal or glucose deprivation. Error bar mean ± s.d. ^*^P < 0.05. ^###^*P* < 0.001, ^##^*P* < 0.01, ^#^*P* < 0.05. **D** Cell apoptosis of MS751 cells with or without MAT2A depletion was analyzed by Annexin V assay followed by flow cytometry (Left panel). Quantitative results were shown in Right panel. Error bar mean ± s.d. ^***^P < 0.001, ^*^P < 0.05. **E**, **F** Cell apoptosis of C33A cells (**E**) and MS751 cells (**F**) with or without PDCD6 depletion was analyzed by Annexin V assay followed by flow cytometry (Left panel). Quantitative results were shown in right panel. Error bar mean ± s.d. ^**^P < 0.01. **G**–**I** Cell apoptosis was analyzed by immunoblotting with the indicated antibodies under normal or glucose deprivation in MS751 cells with or without MAT2A depletion (**G**), C33A cells with or without PDCD6 depletion (**H**) and MS751 cells with or without PDCD6 depletion (**I**).

The CCK-8 and the AnnexinV/PI analyses together indicated that MAT2A depletion led to the impaired growth (Fig. 2A) and enhanced apoptosis (Fig. 2D) in MS751 cells under both normal and glucose deprivation conditions, revealing the promoting effect of MAT2A on growth of cervical cancer cells. In addition, PDCD6 silence by the shRNA resulted in retarded cell growth (Fig. 2B) with an increased apoptotic rate in C33A cells (Fig. 2E), while this effect was aggravated under the condition of glucose deficiency. Similar effect from PDCD6 knockdown group was also observed in MS751 cells (Fig. 2C and F). Moreover, data from Western blotting analysis demonstrated that the expression of cleaved-caspase-3 and cleaved-PARP was increased in sh-MAT2A cells and sh-PDCD6 cells after incubation with glucose free DMEM (Fig. 2G–I). These data indicated that PDCD6 prevented cervical cancer cells from apoptosis and thus maintained cell growth under glucose deprivation.

### MAT2A mediates PDCD6 methylation

**Fig. 3:**
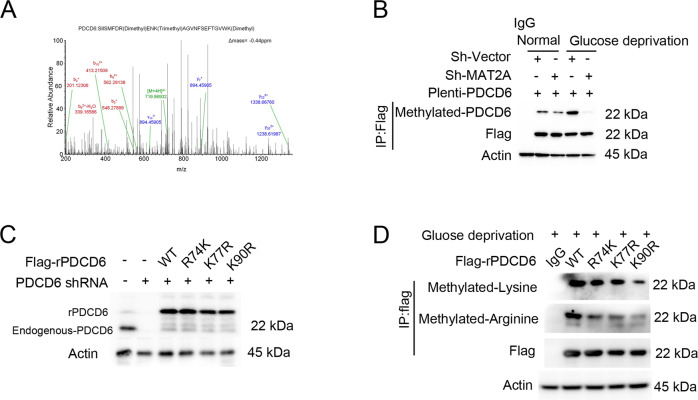
PDCD6 is methylation at lysine 90 and increases the protein stability. **A** MS751 cells with expressing 3×flag-MAT2A were cultured for 12 h under glucose deprivation. Immunoprecipitation was performed using Flag antibody, and the extracts were analyzed by mass spectrometry. The results of a mass spectrometric analysis revealed that 3 amino acids, R74, K77 and K90, of PDCD6 were methylated. **B** MS751 cells transfected with shMAT2A or 3×flag-PDCD6 were cultured for 12 h under normal or glucose deprivation. Cellular extracts were subjected to immunoprecipitation with an anti-methylated PDCD6 antibody. **C** MS751 cells were transfected a vector for control shRNA or PDCD6 shRNA and reconstituted with expression of WT rPDCD6, rPDCD6 R74K, rPDCD6 K77R or rPDCD6 K90R. Cell lysates were directly subjected to immunoblotting with the indicated antibodies. **D** MS751 cells were transfected with a vector for control shRNA or PDCD6 shRNA and reconstituted with expression of WT rPDCD6, rPDCD6 R74K, rPDCD6 K77R or rPDCD6 K90R. Cellular extracts were subjected to immunoprecipitation with an anti-arginine or lysine methylation antibody.

Mass spectrometry analysis of MAT2A precipitates from MS751 cells showed that three amino acids R74, K77 and K90 within PDCD6 were methylated (Fig. 3A). Notably, immunoprecipitation analysis indicated that the methylation level of PDCD6 was increased under glucose deprivation, which was largely blocked by MAT2A depletion (Fig. 3B). This finding revealed that MAT2A was essential for PDCD6 methylation. Subsequently, endogenous PDCD6 was depleted and the RNAi-resistant rPDCD6 WT, rPDCD6 R74K, rPDCD6 K77R or rPDCD6 K90R were transduced in MS751 cells (Fig. 3C). Consequently, expression of rPDCD6 K90R resulted in a further reduction of total methylation signal than that after expression of rPDCD6 R74K, rPDCD6 K77R, indicating that K90 might be the primary methylation site of PDCD6 (Fig. 3D). Of note, rPDCD6 K90R expression also displayed a lower arginine methylation level than the WT counterpart (Fig. 3D), and this implied that PDCD6 K90 methylation would be required for the relevant arginine methylation of protein.

### K90 mutation promotes tumor apoptosis in vitro and in vivo

**Fig. 4:**
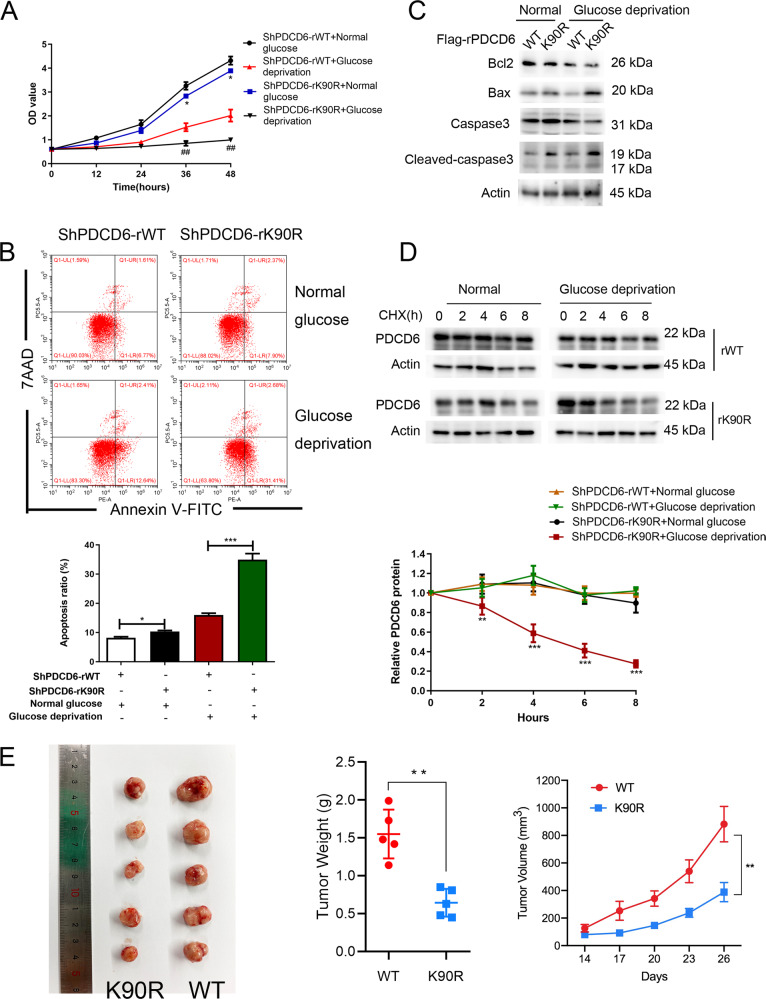
PDCD6 K90 mutation promotes tumor apoptosis in vitro and in vivo. **A** The proliferation of MS751 cells with WT rPDCD6 or rPDCD6 K90R mutation was examined by CCK8 assay under normal or glucose deprivation. ^*^P < 0.05, ^##^*P* < 0.01. **B** Cell apoptosis of MS751 cells with WT rPDCD6 or rPDCD6 K90R mutation was analyzed by Annexin V assay followed by flow cytometry (Top panel). Quantitative results were shown in bottom panel. Error bar mean ± s.d. ^***^P < 0.001, ^*^P < 0.05. **C** MS751 cells with WT rPDCD6 or rPDCD6 K90R mutation were analyzed with immunoblotting with the indicated antibodies under normal or glucose deprivation. **D** MS751 cells with depleted PDCD6 and reconstituted expression of WT rPDCD6 or rPDCD6 K90R were treated with CHX (10 μg/ml) for 8 h under glucose deprivation. The below panel showed relative protein amounts of different groups. Error bar mean ± s.d. of triplicate experiments. ^***^P < 0.001, ^**^P < 0.01. **E** K90R mutation inhibits xenograft tumor growth. Subcutaneous xenograft experiment was performed in nude mice using MS751 rPDCD6 WT and rPDCD6 K90R stable cell lines. Diameters of tumors were measured and tumor volumes were calculated. ^**^P < 0.01.

To further determine the physiological impact of methylation of PDCD6 K90, the effect of rPDCD6 K90R expression on cell proliferation and apoptosis was examined. Evidently, rPDCD6 K90R expression slightly suppressed cell proliferation (Fig. 4A) and increased the apoptotic rates (Fig. 4B) in MS751 cells under normal condition in comparison with the WT counterparts. The effect above was greatly aggravated under the condition of glucose deficiency. Consistent with these observations, immunoblotting analysis revealed that expression of rPDCD6 K90R led to a decreased level of Bcl-2 and an apparent induction of pro-apoptotic factors Bax and cleaved-caspase 3 (Fig. 4C). Meanwhile, it was found that the half-life of rPDCD6 K90R protein was largely shortened compared with that of WT rPDCD6 under glucose deprivation (Fig. 4D). These data suggested that MAT2A facilitated PDCD6 methylation, thereby protecting cervical cancer cells from apoptosis under glucose deficiency. To determine the effect of PDCD6 K90 methylation on tumor growth, we performed xenograft experiments using rPDCD6 WT and rPDCD6 K90R stable cell lines. Tumor growth was continuously monitored twice a week. Results demonstrated that MS751 cells expressing K90R mutants displayed a lower growth rate than wild type cells in vivo (Fig. 4E). Similarly, mice injected with K90R mutant cells developed smaller tumor weight than those wild type cells (Fig. 4E). These results suggested that PDCD6 K90R inhibits tumor growth.

### MAT2A and PDCD6 are highly expressed and correlated with poor prognosis in cervical cancer

**Fig. 5:**
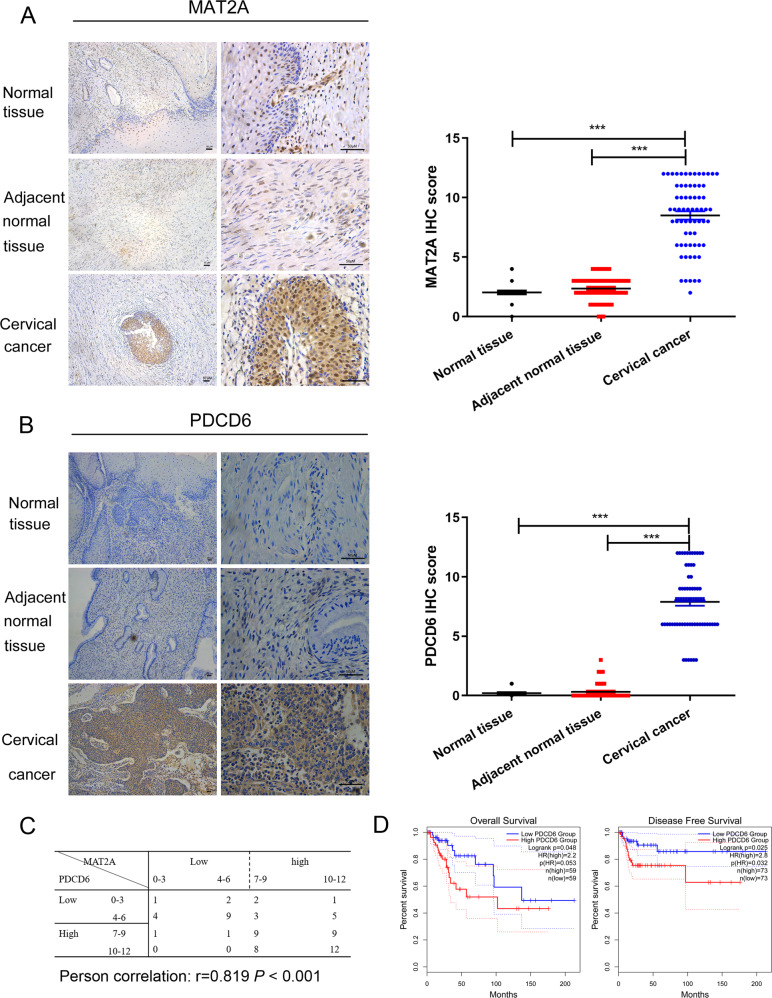
MAT2A and PDCD6 are highly expressed in cervical cancer tissues and are correlated with poor prognosis with cervical cancer patients. **A** Immunohistochemical staining with MAT2A antibody was performed in 67 cervical cancer specimens and 67 normal cervical specimens. Representative photos of tumors versus the normal tissue and adjacent normal tissues were shown (magnification: ×100 and ×400, left panel). Comparative analysis of MAT2A expression among normal cervical tissue, adjacent normal tissue and cervical cancer specimen was shown (right panel).^***^P < 0.001. Scar bars: 50 μM. **B** Immunohistochemical staining with PDCD6 antibody was performed in 67 cervical cancer specimens and 67 normal cervical specimens. Representative photos of tumors versus the normal tissue and adjacent normal tissue was shown (magnification: ×100 and ×400, left panel). Comparative analysis of PDCD6 expression among normal cervical tissue, adjacent normal tissue and cervical cancer specimen was shown (right panel). ^***^P < 0.001. Scar bars: 50 μM. **C** Semiquantitative scoring and correlation analysis indicating the correlation between MAT2A and PDCD6 (r = 0.819, *P* < 0.001). **D** Prognostic analysis of PDCD6 gene expression in cervical cancer patients (GEPIA2) was illustrated. Overall survival (OS) and disease-free survival (DFS) analysis were performed to show the survival status in the TCGA cohort via GEPIA2. Kaplan–Meier curves were plotted with *P*-values and HRs by log-rank tests and Cox regression models.

To examine the clinical relevance of the relationship between MAT2A and PDCD6, 67 pairs of cervical cancer tissues and matched normal cervical tissue specimens were collected for IHC analyses. Results showed that both MAT2A (Fig. 5A) and PDCD6 (Fig. 5B) displayed an upregulated expression in tumor tissues compared with both normal tissue and adjacent normal tissues. Correlation analysis revealed a positive relationship between MAT2A and PDCD6 levels (Fig. 5C). Moreover, Kaplan analysis showed that high levels of PDCD6 expression in cervical cancer specimens were correlated with decreased overall survival and disease-free survival durations of the patients via GEPIA database (Fig. 5D). Twenty-seven tissues of cervical cancer patients were pathologically confirmed as low PDCD6 expression while 40 cases were confirmed as high PDCD6 expression. The characteristics of the two groups were summarized in Table 1. The levels of PDCD6 protein were positively correlated with advance clinical stage of cervical cancer, while no significant differences were obtained in other characteristics between the two groups. These results revealed that high expression of PDCD6 protein potentially contributes to cervical cancer development at late stage.

**Table 1:** Clinical characteristics of cervical cancer patients with PDCD6 expression.

Parameter	Low *N* = 27	High *N* = 40	*P*-Value
Age (years)	52.5(31,81)	50(24,82)	0.623
BMI	22.85(18.2,30.5)	22.3(17.7,30.9)	0.211
Tumor size			0.58
<2 cm	9	16	
2–4 cm	18	24	
Type of surgery			0.932
Open	20	30	
Laparoscopic	7	10	
FIGO stage			0.0001
≤II	27	18	
>II	0	22	

## Discussion

**Fig. 6:**
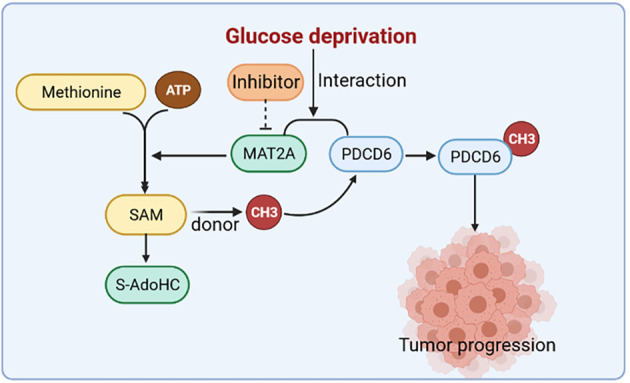
The schematic model showing the interaction between MAT2A and PDCD6 under glucose deprivation in cervical cancer cells. MAT2A facilitates PDCD6 methylation to promote cervical cancer growth under glucose deprivation.

Cancer cells face with multiple metabolic stress conditions like deficient nutrients or oxygen. Starvation of glucose is one of the major forms of metabolic stress in cancer cells [19, 22]. The survival and proliferation of cancer cells are highly dependent on glucose or glutamine [23]. Numerous studies have established that glycogen metabolism is vital for cancer metabolism [24, 25]. However, it is largely unclear how cervical cancer cell metabolism is regulated under glucose deficiency. In this study, we found that glucose deprivation treatment largely enhanced MAT2A-PDCD6 complex formation in cervical cancer cells, which can be promoted by AMPK pathway (Fig. 6). AMPK, a fuel-sensing enzyme, is activated under energy deficiency and is suppressed in energy surplus. To cope with intense energy demands, AMPK is activated to stimulate the fatty acid oxidation and inhibit ATP consumption during anabolism [26]. Herein, we further demonstrated that under glucose deprivation, knockdown of MAT2A would inhibit PDCD6 activity, and this change was reversed by the protease inhibitor MG132 treatment. Consistently, MAT2A depletion was found to display a shortened half-life of PDCD6 under glucose deprivation stress. It is suggested that MAT2A is required for PDCD6 protein stability maintenance.

PDCD6 is known as apoptosis-linked gene-2 (ALG-2), which is associated with cell proliferation and cell apoptosis [27]. PDCD6 has not been defined as an oncogene or tumor suppressor as it has opposing effects in different types of tumors [28, 29]. In our study, we demonstrated that MAT2A facilitates PDCD6 methylation that is critical for PDCD6 protein stability maintenance under glucose deficiency, and thereby suppresses cell apoptosis of cervical cancer cells. Previous studies have demonstrated that PDCD6 is involved in apoptosis induced by T-cell receptor, Fas and glucocorticoid, as well as endoplasmic reticulum stress in the process of organ formation [12, 13]. Thus, our findings support an anti-apoptotic role of PDCD6 in cervical cancer cells at glucose deficiency condition and highlight the positive relationship between PDCD6 and MAT2A during cervical cancer development. Lysine methylation acts as key regulatory factor in epigenetic control of gene function, and plays a vital role of protein-protein binding [30]. Moreover, K90 site is identified as the primary methylation of PDCD6 under glucose deficiency, which is critical for PDCD6 protein stability and the relevant physiological events. However, the molecular mechanism of PDCD6 K90 methylation occurrence and how it regulates protein stabilization are worthy of further investigation.

In line with the results from in vitro study, the further clinical analysis indicates the MAT2A level is positively related to PDCD6 level in cervical cancer tissues. Further analyses showed that a high level of PDCD6 was enriched in tumors with late stage. These results revealed the close relationship between MAT2A level and cervical malignancy, suggesting that PDCD6 expression may play a critical role in tumorigenesis of human cervical cancer. Taken together, our results revealed that MAT2A facilitates methylation of PDCD6 at K90 site to promote cervical cancer growth under glucose deprivation mediated by AMPK activation. These results reveal the close relationship between MAT2A level and cervical malignancy and demonstrate the critical role of PDCD6 in cervical tumorigenesis.

## Materials and methods

### Reagents

Anti-MAT2A antibody (NBP1-92100, Rabbit polyclonal) was purchased from Novus Biologicals Company. Anti-PDCD6 antibody (12303-1-AP, Rabbit polyclonal) was purchased from Proteintech Company. Antibodies against Methylated-Lysine (ab23366, Rabbit polyclonal) and Pro-Caspase (ab32150, Rabbit monoclonal) were obtained from Abcam Company. Rabbit polyclonal antibody against Dimethyl-Arginine (07-414) was purchased from Millipore Sigma Company. Monoclonal antibody (F1804) that recognizes Flag was purchased from Sigma. Antibodies against β-actin (3700), Bcl-2 (15071), Bax (5023), Cleaved-Caspase 3 (9664), PARP (9532), Cleaved-PARP (5652), p-Acc (11818), p-JunB (8053) and p-MAPKAPK2 (3044) were purchased from Cell Signaling Technology. AMPK inhibitor compound C, SP600125 and SB203580 were obtained from Selleck Company. Cell counting kit 8 (CCK-8) was purchased from BestBio Science (Beijing, China). Annexin V-FITC/7-AAD was obtained from BD (Pharmingen, CA, USA). All other chemicals were obtained from commercially available. MG132 (protease inhibitor) and cycloheximide (CHX) were purchased from Sigma-Aldrich (USA).

### Mass spectrometric analysis

MAT2A-associated proteins were analyzed by LC-MS/MS [31]. Briefly, 3×Flag-MAT2A associated proteins from the immune-precipitation assay were acetone-precipitated at −20 °C overnight and resuspended in 50 mM ammonium bicarbonate buffer containing Rapigest (Waters Corp). After the samples were heated at 95 °C for 10 min and then cooled down, 100 ng trypsin (Promega) was added. The digestion was maintained at 37 °C overnight and then analyzed by a mass spectrometer (Thermo Fisher Scientific). Proteins were analyzed by Sequest v.1.20 via Proteome Discoverer v.1.3 software.

### Cell culture

All of the cell lines were obtained from Type Culture Collection of the Chinese Academy of Medical Science (Shanghai, China) and routinely tested for mycoplasma contamination. Normal cervical epithelial cells (ECT1) and cervical cancer cells (HeLa, C33A and MS751) were grown in Dulbecco’s modified Eagle’s medium (DMEM/high-glucose supplemented) with 10% fetal bovine serum (FBS, Gibco, USA). CaSki and SiHa cells were maintained in RPMI 1640 medium plus 10% FBS. Cells were grown under an atmosphere with 5% CO_2_ at 37 °C. To induce glucose deficiency, cells were incubated with glucose-free DMEM or RPMI 1640 medium containing 10% FBS. The cells were pretreated with the corresponding inhibitors, Compound C (10 μM), SP600125 (20 μM) and SB203580 (10 μM) for 1 h before being cultured with glucose deprivation for 12 h. Cell lysates were directly subjected to western blotting analysis.

### DNA constructs and mutagenesis

PCR-amplified human MAT2A was generated with pLenti-3×Flag vector. Human PDCD6 was cloned into pLenti-3×Flag vector. Flag-PDCD6 R74K, Flag-PDCD6 K47R, Flag-PDCD6 K90R were constructed using the QuikChange site-directed mutagenesis kit (Stratagene). Human pGIPZ-MAT2A shRNA was generated with the oligonucleotide 5’-AGG TTT TGA CTA CAA GAC T-3’, human pGIPZ-PDCD6 shRNA was generated with the ologonucleotide 5’-CTG CAG AGG TTG ACG GAT A-3'. The pGIPZ control was generated with control oligonucleotide 5’-GCT TCT AAC ACC GGA GGT CTT-3’.

### Transfection

MS751 and C33A cells were transfected with various plasmids using lipofectamine 2000 (Invitrogen) according to the vendor’s instructions. Lentiviral constructs were transfected together with psPAX2 and PMD2.G into HEK293 cells. After 48 h transfection, viral supernatant was infected into targeting cells.

### Immunoprecipitation and immunoblotting analysis

Proteins were extracted from cultured cells using a modified buffer (50 mM Tris-HCl (pH 7.5), 1% Triton X-100, 150 mM NaCl, 0.5 mM EDTA, 1 mM dithiothreitol, and protease inhibitor cocktail and phosphatase inhibitor cocktail), followed by immunoprecipitation and immunoblotting with the corresponding antibodies. The protein concentration was determined using the Bradford assay. Protein from cellular lysates was separated by 12% SDS-PAGE and transferred onto PVDF membrane. The membranes were blocked with 5% non-fat mike for 2 h at room temperature and probed with the indicated antibodies at 4 °C overnight. The membranes then were probed with secondary antibody and visualized by Enhanced Chemiluminescence (ECL) substrate using Amersham Imager 600 system.

### Quantitative RT-PCR

Total RNA was isolated with TRIzol agent using standard techniques, and reverse transcription of RNA was performed using a Revert Transcription Kit followed by the manufacturer’s instructions (Thermo Scientific, Wilmington, USA). Then the cDNA was synthesized using SYBR Green qPCR Master Mix and conducted on a Light cycler 480 (Roche, France). The expression of genes was normalized to the GAPDH gene using the 2^−△△Ct^ method. All transcripts were independently quantified for three times. Primer sequences were as follows: PDCD6 (sense), 5’-CCG TCT CTC CCA GCC TTC TC-3’ and PDCD6 (reverse), 5’-CTC CCA CCA CCA CCA CAA AC-3’, GAPDH (sense), 5’-GAA GGT GAA GGT CGG AGT C-3’ and GAPDH (reverse), 5’-GAA GAT GGT GAT GGG ATT TC-3’.

### Determination of PDCD6 protein stability

MS751 cells were transfected with pGIPZ control and pGIPZ-MAT2A shRNA or Flag-PDCD6 K90R. The cells were treated with CHX at the final concentration of 10 μg/mL. Cell lysates were subsequently applied for different time course (0, 2, 4, 6, 8 h) and were used for western blotting analysis. For experiment using MG132, after the cells transfected with pGIPZ-MAT2A shRNA were co-treated with 10 μM MG132 for 6 h, the cell lysates were prepared and analyzed by western blotting analysis.

### Cell viability analysis

CCK-8 assay was used to determine the cell viability. A total of 5 × 10^4^ cells per well were plated in 96-well plates with 100 μL media. After incubation overnight, the medium was replaced with DMEM/high-glucose or DMEM/glucose-free for incubation in the following 12, 24 and 48 h. Finally, 10 μL of CCK-8 reagent was added to each well and incubated at 37 °C for 2 h before measuring at a wavelength of 450 nm by a microplate reader (Bio-Rad).

### Flow cytometry analysis

Cells were plated at 5 × 10^5^ cells/dish into 60 mm dishes. After appropriate treatment and incubation, cells were collected with 0.25% trypsin, washed with cold PBS for twice, and stained using Annexin V-FITC/propidium iodide or PE/7-AAD for 15 min in dark at room temperature according to the manufacturers’ protocol. The rate of apoptotic cells was recorded using flow cytometry (BD Pharmingen USA). The experiment was repeated three times.

### Immunohistochemical (IHC) staining

Human cervical cancer samples and normal cervical samples were obtained from The Second Affiliated Hospital of Wenzhou Medical University from 2009 to 2019 after surgical resection. All samples used in this research were obtained with written informed consent from patients. The procedures related to human subjects were approved by the Ethics Committee of the Institutes of The Second Affiliated Hospital of Wenzhou Medical University. IHC was performed on paraffin-embedded sections of these tissues after fixation. The sections were microwaved for antigen retrieval and incubated with MAT2A (1:500) and PDCD6 (1:500) primary antibodies overnight at 4 °C. After incubation with the primary antibody, the sections were washed and incubated with secondary antibodies and DAB staining reagent kit (GTVision Detection System/Mo&Rb kit) according to the manufacturer’s instruction. The results were scored by two independent authors blinded to the clinicopathologic data. The tissue sections were quantitatively scored according to the staining intensity (0, no signal; 1, weak; 2, moderate; and 3, strong) and percentage of positive cells (1, 0–25%; 2, 26–50%; 3, 51–75%; and 4, 76–100%). These two scores were then multiplied, resulting in a score ranged from 0 to 12. The specimens with score >6 were classified as high expression while those with score ≤6 were classified as low expression [31].

### GEPIA database analysis

The GEPIA database (http://gepia2.cancer-pku.cn) is a public database used to analyze differences of gene expression in cancer and normal tissues, including 9736 tumors and 8587 normal samples from TCGA and GTEX databases [32]. We performed correlative prognostic analysis of PDCD6 via “Single Gene Analysis” module. The *P* value was 0.05, and the Kaplan-Meier curve was used to analyze the prognosis.

### In vivo experiment

Ten female six weeks old nu/nu mice were divided into two groups (five mice per group). Subsequently, 2 × 10^6^ RIPA-knockout MS751 cells with rPDCD6 WT and rPDCD6 K90R were subcutaneously injected in the left and right flanks, respectively. Tumor length (a) and width (b) were measured using vernier calipers twice a week, Tumor volume was calculated using the equation: V = ab^2^/2. After 30 days, mice were sacrificed and tumor mass were weighted and photographed. All mice were housed under standard lighting (12 h light/dark cycle) and temperature (23 ± 1 ^o^C) conditions, with free access to nutritionally balanced diet and deionized water. Animals were maintained in accordance with institutional guidelines for care and use of laboratory animals. Protocols were reviewed and approved by committee on Animal Research of Wenzhou Medical University.

### Statistical analysis

Data were represented the mean ± standard deviation (SD). Statistical differences were conducted with two-tailed unpaired Student’s t test and the χ^2^ test. Correlation analysis of the expression of MAT2A and PDCD6 was conducted using Spearman’s test. Fisher’s exact and χ^2^ tests were used to compare categorical proportions between the low and high expression of PDCD6 groups. SPSS 22.0 and GraphPad Prism 6.0 software were conducted in the analyses. *P* < 0.05 was considered statistically significant.

## Supplementary information

Original WB images

## Data Availability

The data in this study are available from the corresponding author on reasonable request.
