# Supplementary material for: MAT2A facilitates PDCD6 methylation and promotes cell growth under glucose deprivation in cervical cancer
Source: Cell Death Discov. 2022 Apr 8;8:176. doi: 10.1038/s41420-022-00987-6 (PMC8993843; doi:10.1038/s41420-022-00987-6)
Supplement: Supplementary file 1 — Original WB images [file 41420_2022_987_MOESM1_ESM.pdf]

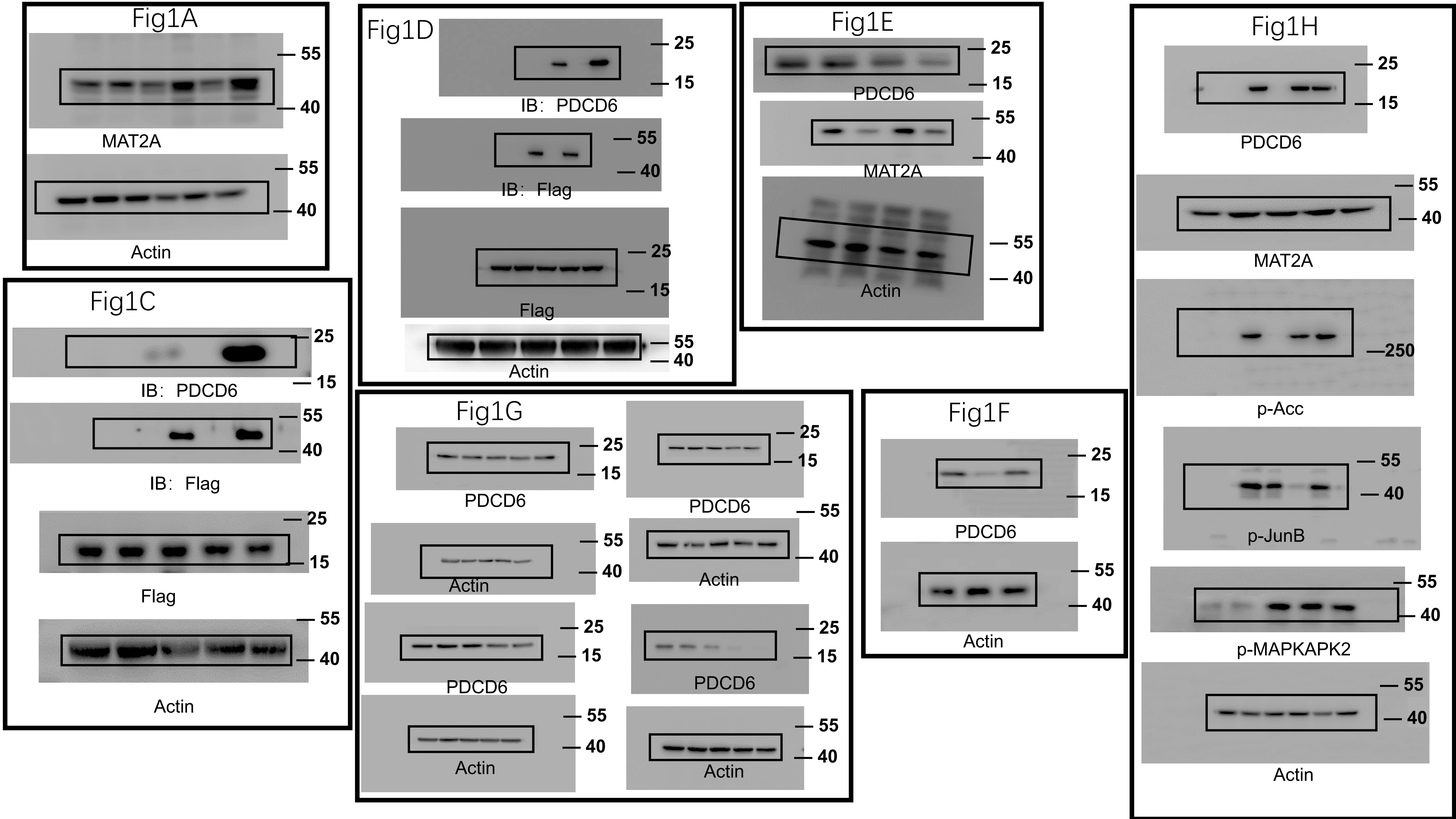
Fig1A
MAT2A
Actin
55
40
Fig1C
IB: PDCD6
IB: Flag
Flag
Actin
25
15
55
40
Fig1D
IB: PDCD6
IB: Flag
Flag
Actin
Fig1E
PDCD6
MAT2A
Actin
Fig1F
PDCD6
Actin
Fig1G
PDCD6
Actin
Fig1H
PDCD6
MAT2A
p-Acc
250
p-JunB
p-MAPKAPK2
Actin

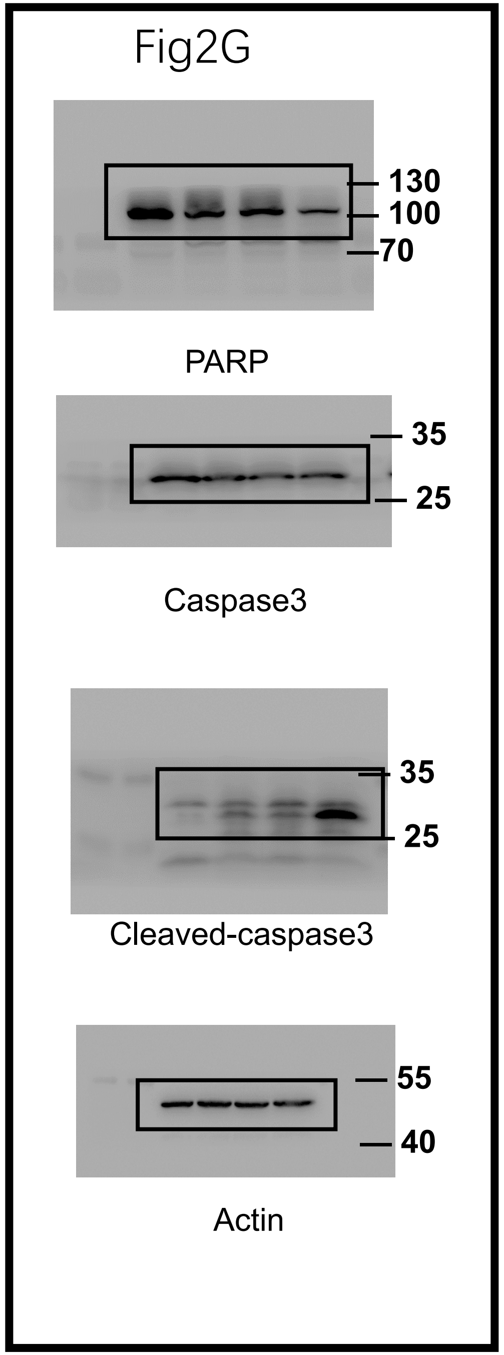
Fig2G
130
100
70
PARP
35
25
Caspase3
35
25
Cleaved-caspase3
55
40
Actin

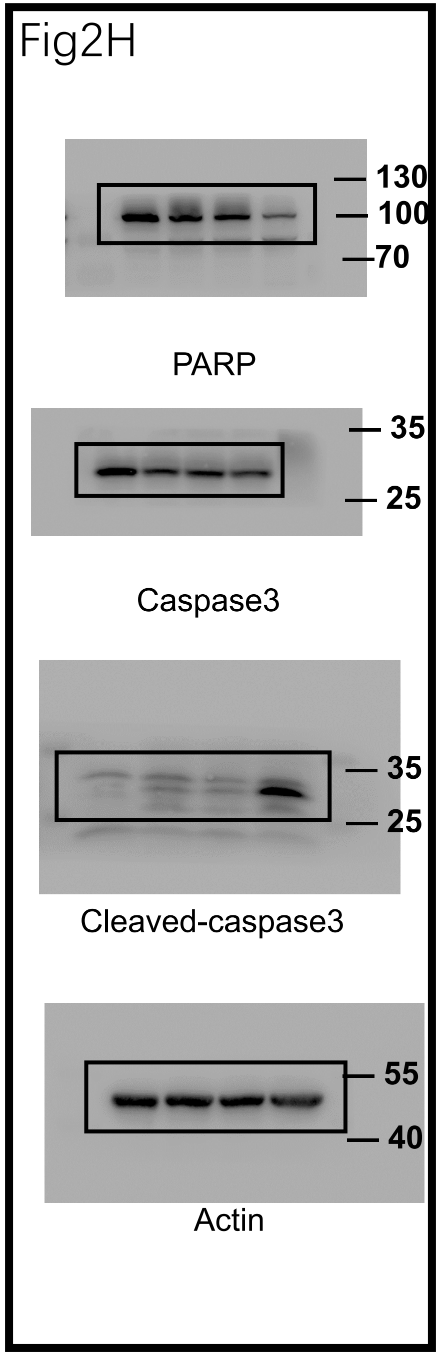
Fig2H
130
100
70
PARP
35
25
Caspase3
35
25
Cleaved-caspase3
55
40
Actin

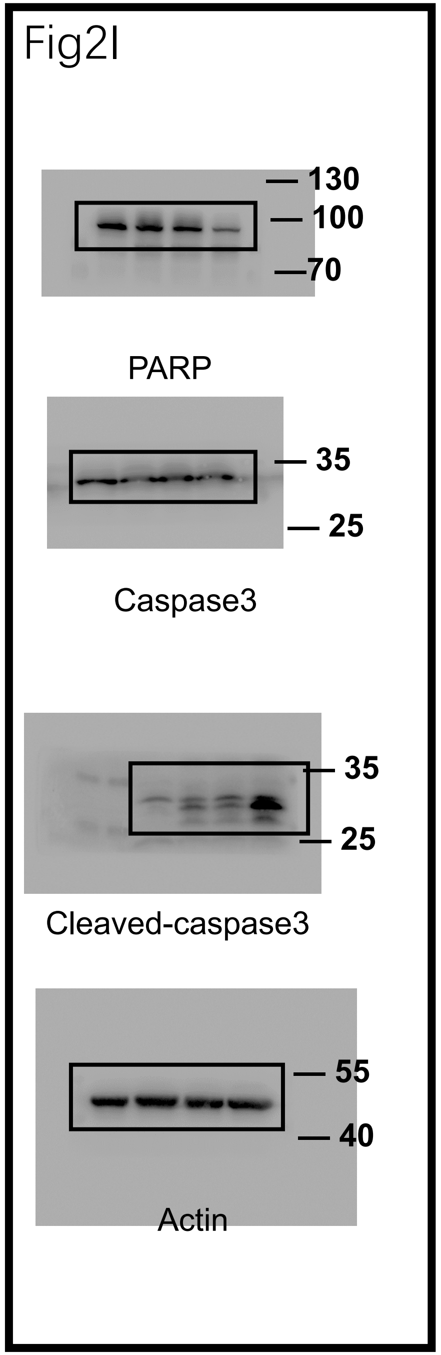
Fig2I
130
100
70
PARP
35
25
Caspase3
35
25
Cleaved-caspase3
55
40
Actin

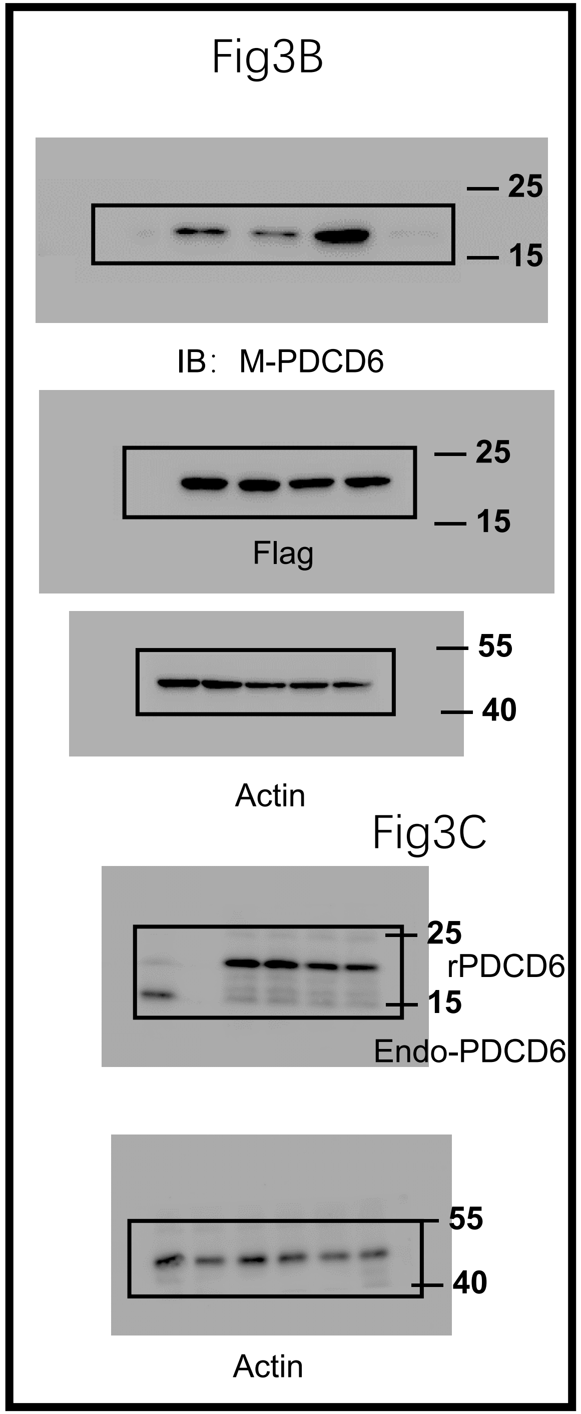
Fig3B
25
15
IB：M-PDCD6
25
15
Flag
55
40
Actin
Fig3C
25
rPDCD6
15
Endo-PDCD6
55
40
Actin

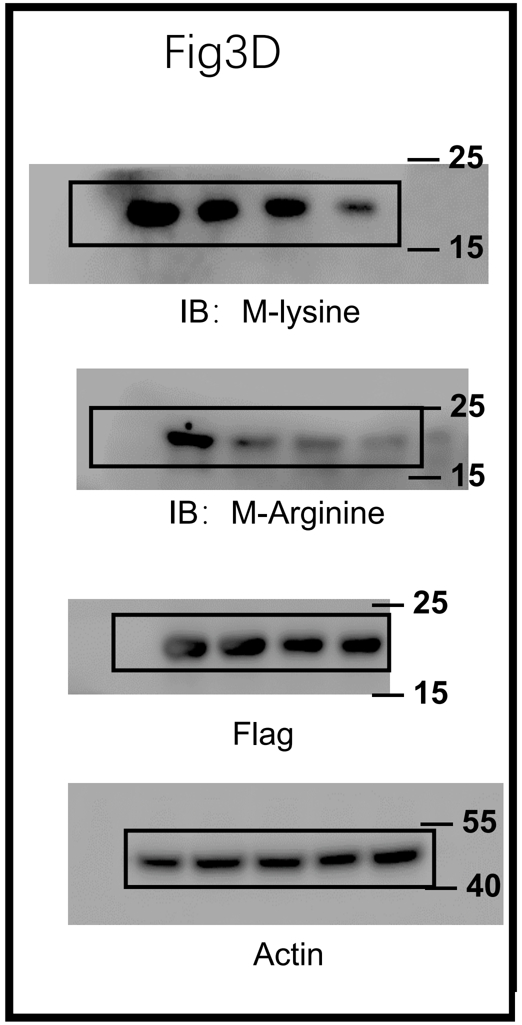
Fig3D
25
15
IB：M-lysine
25
15
IB：M-Arginine
25
15
Flag
55
40
Actin

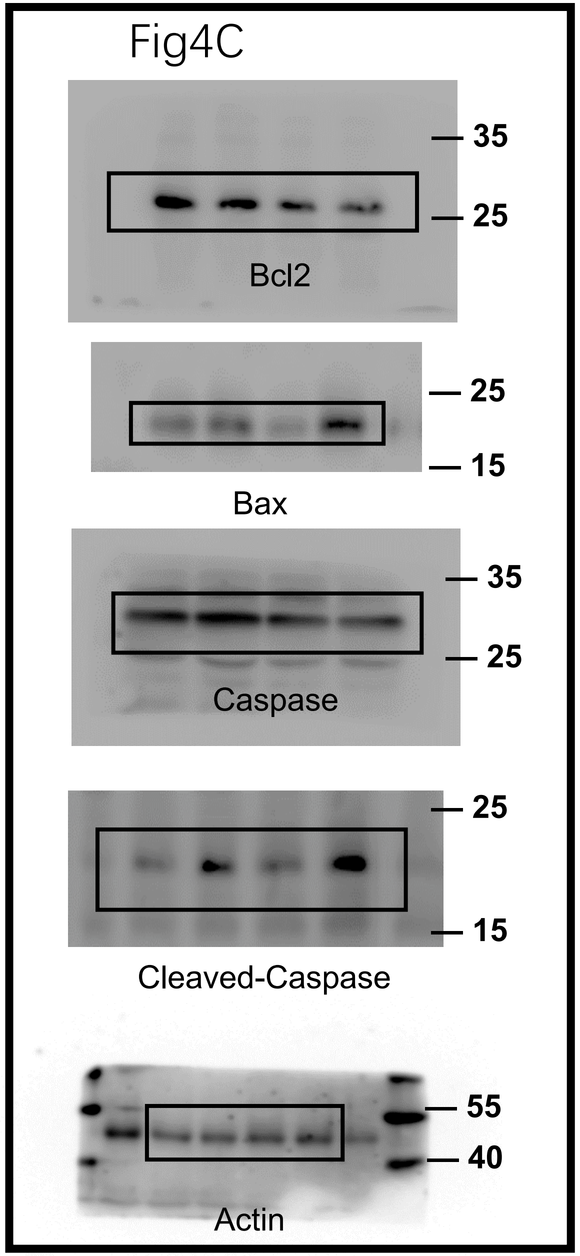
Fig4C
35
25
Bcl2
25
15
Bax
35
25
Caspase
25
15
Cleaved-Caspase
55
40
Actin

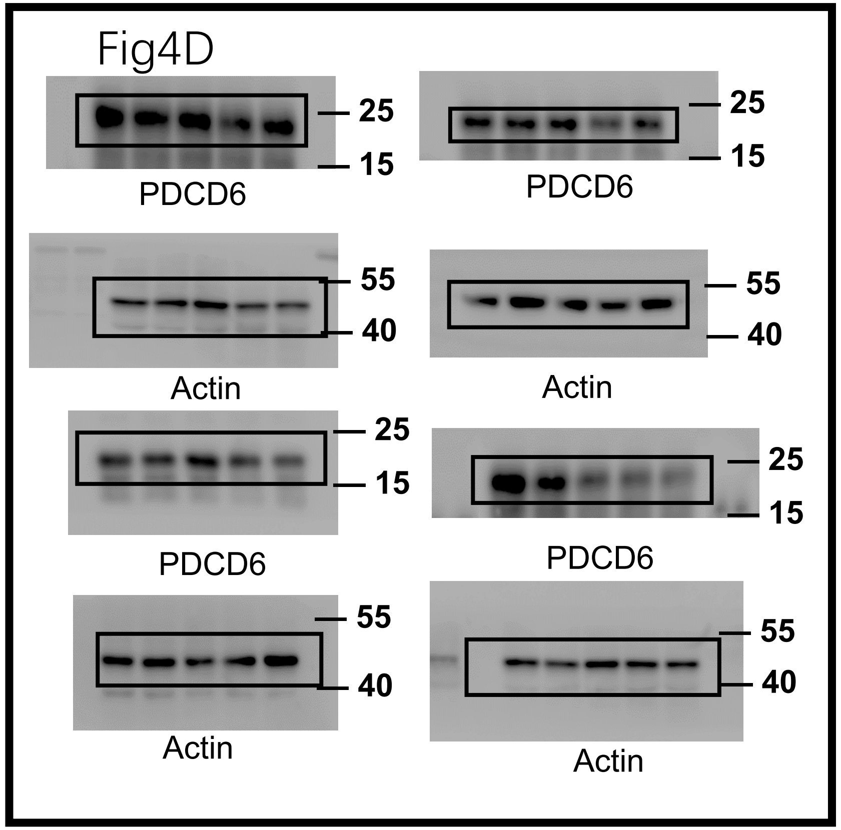
Fig4D
25
15
PDCD6
25
15
PDCD6
55
40
Actin
55
40
Actin
25
15
PDCD6
25
15
PDCD6
55
40
Actin
55
40
Actin
